# Supplementary figures and images for: Simultaneous rapid detection of Hantaan virus and Seoul virus using RT-LAMP in rats
Source: PeerJ. 2019 Jan 8;6:e6068. doi: 10.7717/peerj.6068 (PMC6329334; doi:10.7717/peerj.6068)

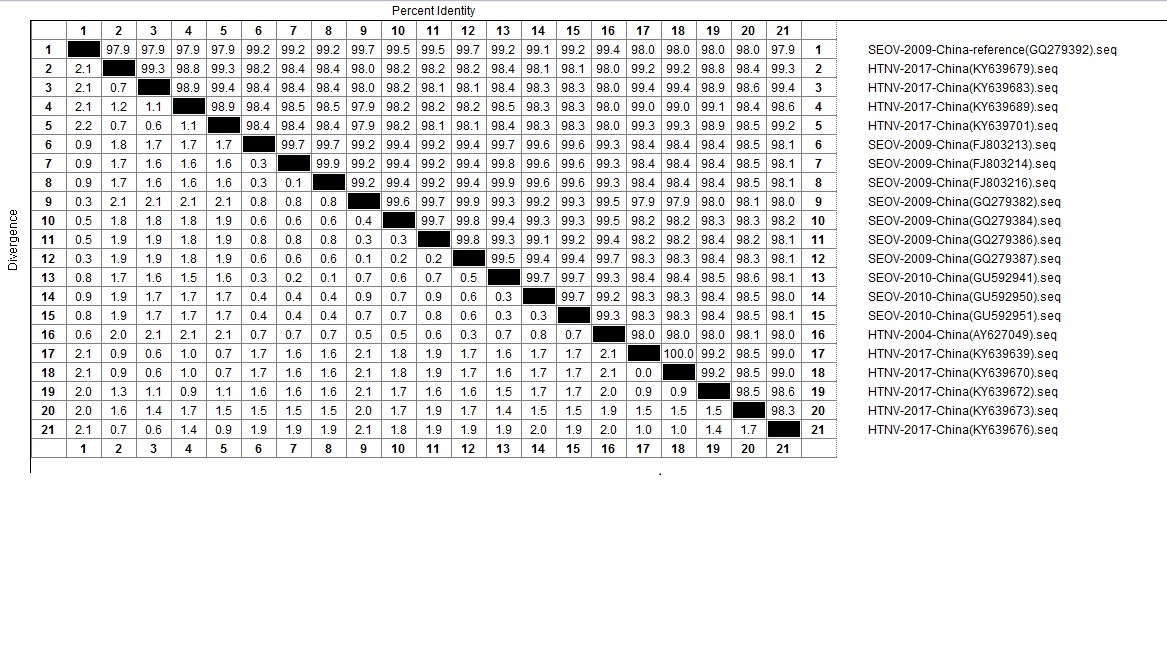

Supplement: Supplemental Information 1 [file peerj-07-6068-s001.jpg]

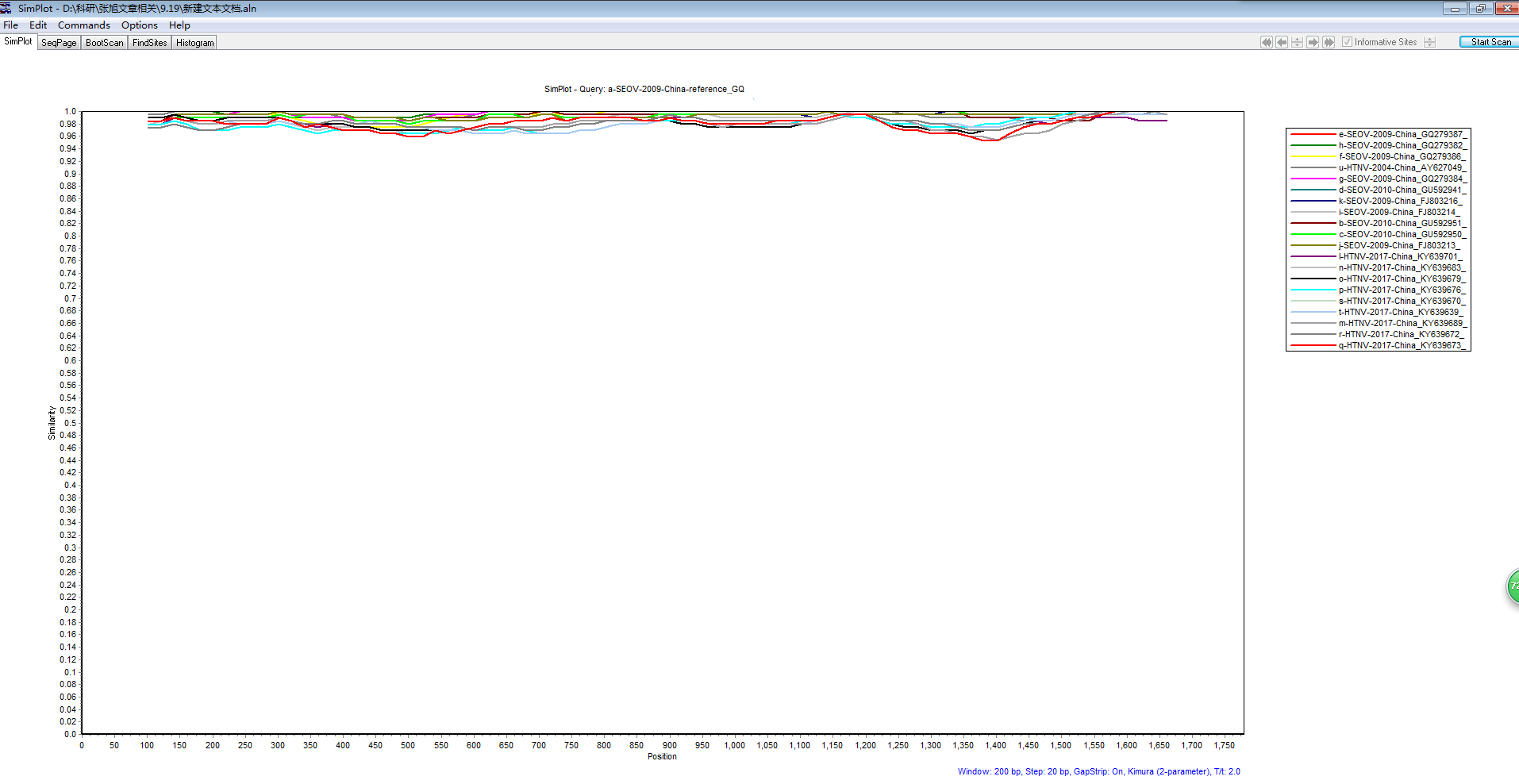

Supplement: Supplemental Information 2 [file peerj-07-6068-s002.png]
