# Supplementary material for: Simultaneous rapid detection of Hantaan virus and Seoul virus using RT-LAMP in rats
Source: PeerJ. 2019 Jan 8;6:e6068. doi: 10.7717/peerj.6068 (PMC6329334; doi:10.7717/peerj.6068)
Supplement: Supplemental Information 7 [file peerj-07-6068-s007.docx]

S gene nucleotide sequences of reference strains (GQ279392) and other strains from China.

**SEOV-2009-China-reference (GQ279392)**

tagtagttgactccctaaagagctattacactaacaagaaaaatggcaactatggaagaaatccagagagaaatcagtgctcacgaggggcagcttgtgatagcacgccagaaggtcaaggatgcagaaaagcagtatgagaaggatcctgatgacttaaacaagagggcactgcatgatcgggagagtgttgcagcttcaatacaatcaaaaattgatgaattgaagcgccaacttgccgacaggattgcagcagggaagaacatcgggcaagaccgggatcctacaggggtagagccgggtgatcatctcaaggaaagatcagcactaagctatgggaatacactggacctgaatagtcttgacattgatgaacctacaggacagacagctgattggttgactataattgtctatctgacatcattcgtggtcccaatcatcttgaaggcactgtacatgttaacaacaagaggtaggcagacttcaaaggacaacaagggaatgaggatcagattcaaggatgacagctcatatgaggatgtcaatggaatcagaaagcccaaacatctgtatgtgtcaatgccaaatgcccaatcaagcatgaaagctgaagagataacacctggaagattccgcacggcagtatgtgggttatatcctgcacagataaaggcaaggaacatggtaagccctgtcatgagtgtagttgggtttttggcactggcaaaagactggacatctagaattgaagaatggcttggcgcaccctgcaagttcatggcagagtctcccattgccgggagtttatctgggaatcctgtaaatcgtgactatatcagacagagacaaggtgcacttgcaggaatggagccaaaggaatttcaagccctcaggcaacatgcaaaggatgctggatgtacactggttgaacatattgagtcaccatcgtcaatatgggtgtttgctggggcccctgataggtgtccaccaacatgcttgtttgtcggagggatggctgaattaggtgccttcttttctatccttcaggacatgaggaacacaatcatggcttcaaaaactgtgggcacagctgatgaaaagcttcgaaagaaatcatcattctatcaatcatacctcagacgcacacaatcaatgggaatacaactggaccagaggataattgttatgtttatggttgcctggggaaaggaagcagtggacaactttcatctcggtgatgacatggatccagagcttcgtagcctggctcagatcctgattgaccagaaagtgaaggaaatctctaaccaggaacctatgaaactataagtacataattatgtaatcaatactaactataggttaagaaatactaatcattagttaataaggatacagatttattgactaatcatattaaataattaggtaagttaaccattatttagttaagttagctaattgatttatatgattatcacaattgaatgtaatcataagcacaatcactgccatgtataatcacgggtatacgggtggttttcatatggggaacagggtgggcttagggccaggtcaccttaagtgacctttttttgtatatatggatgtagatttcaattgatcgaatactaatcctactgtcctcttttcttttcctttctccttctttactaacaacaacaaactacctcacaaccttctacctcaatatatactacctcattaagttgtttccttttgtctttttagggagcatacttactact

**SEOV-2010-China (GU592951)**

tagtagtagactccctaaaaagctactacactaacaagaaaaatggcaactatggaagaaatccagagagaaatcagtgctcacgaggggcagcttgtgatagcacgccagaaggtcaaggatgcagaaaagcagtatgagaaggatcctgatgacttaaacaagagggcactgcatgatcgggagagtgtcgcagcttcaatacaatcaaaaattgatgaattgaagcgccaacttgccgacaggattgcagcagggaagaacatcgggcaagaccgggatcctacaggggtagagccgggtgatcatctcaaggaaagatcagcactaagctatgggaatacactggacctgaatagtcttgacattgatgaacctacaggacagacagctgattggttgaccataattgtctatctgacatcattcgtggtcccaatcatcttgaaggcactgtacatgttaacaacaagaggtaggcagacttcaaaggacaacaaggggatgaggatcagattcaaggatgacagctcatatgaggatgtcaatggaatcagaaagcccaaacatctgtatgtgtcaatgccaaacgcccaatcaagcatgaaagctgaagagataacacctggaagattccgcacggcagtatgtgggttatatcctgcacagataaaggcaaggaacatggtaagccctgtcatgagtgtagttgggtttttggcactggcaaaagactggacatctagaattgaagaatggcttggcgcaccctgcaagttcatggcagagtctcccattgccgggagtttatctgggaatcctgtgaatcgtgactatatcagacagagacaaggtgcacttgcaggaatggagccaaaggaatttcaagccctcaggcaacatgcaaaggatgctggatgtacactggttgaacatattgagtcaccatcgtcaatatgggtgtttgctggggcccctgataggtgtccaccaacatgcttgtttgtcggagggatggctgagttaggtgccttcttttctatccttcaggacatgaggaacacaatcatggcttcaaaaactgtgggcacagctgatgaaaagcttcgaaagaaatcatcattctatcaatcatacctcagacgcacacaatcaatgggaatacaactggaccagaggataattgttatgtttatggttgcctggggaaaggaagcagtggacaactttcatctcggtgatgacatggatccagagcttcgtagcctggctcagatcctgattgaccagaaagtgaaggaaatctctaaccaggaacctatgaaactataagtacataattatgtaatcaatactaactataggttaagaaatactaatcattagttaataaggatacagatttattgactaatcatattaaataattaggtaagttaactattatttagttaagtttgctaattgatttatatgattatcacaattgaatgtaatcataagcacaatcactgccatgtataatcacgggtatacgggtggttttcatatggggaacagggtgggcttagggccaggttaccttaagtgacctttttttgtatatatggatgtagatttcaattgatcgaatactaatcctactgtcctcttttcttttcctttctccttctttactaacaacaacaaactacctcacaaccttctacctcaatatatactacctcattaagttgtttccttttgtctcttttagggagcatactactacta

**SEOV-2010-China (GU592950)**

tagtagtaggctccctaaaaagctactacactaacaagaaaaatggcaactatggaagaaatccagagagaaatcagtgctcacgaggggcagcttgtgatagcacgccagaaggtcaaggatgcagaaaagcagtatgagaaggatcctgatgacttaaacaagagggcactgcatgatcgggagagtgtcgcagcttcaatacaatcaaaaattgatgaattgaagcgtcaacttgccgacaggattgcagcagggaagaacatcgggcaagaccgggatcctacaggggtagagccgggtgatcatctcaaggaaagatcagcactaagctatgggaatacactggacctgaatagtcttgacattgatgaacctacaggacagacagctgattggttgaccataattgtctatctgacatcattcgtggtcccaatcatcttgaaggcactgtacatgttaacaacaagaggtaggcagacttcaaaggacaacaaagggatgaggatcagattcaaggatgacagctcatatgaggatgtcaatggaatcagaaagcccaaacatctgtatgtgtcaatgccaaacgcccaatcaagcatgaaagctgaagagataacacctggaagattccgcacggcagtatgtgggttatatcctgcacagataaaggcaaggaacatggtaagccctgtcatgagtgtagttgggtttttggcactggcaaaagactggacatctagaattgaagaatggcttggtgcaccctgcaagttcatggcagagtctcccattgccgggagtttatctgggaatcctgtgaatcgtgactatatcagacagagacaaggtgcacttgcaggaatggagccaaaggaatttcaagccctcaggcaacatgcaaaggatgctggatgtacactggttgaacatattgagtcaccatcgtcaatatgggtgtttgctggggcccctgataggtgtccaccaacatgcttgtttgtcggagggatggctgagttaggtgccttcttttctatccttcaggacatgaggaacacaatcatggcttcaaaaactgtgggcacagctgatgaaaagcttcgaaagaaatcatcattctatcaatcatacctcagacgcacacaatcaatgggaatacaactggaccagaggataattgttatgtttatggttgcctggggaaaggaagcagtggacaactttcatctcggtgatgacatggatccagagcttcgtagcctggctcagatcctgattgaccagaaagtgaaggaaatctctaaccaggaacctatgaaactataagtacataattatgtaatcaatactaactataggttaagaaatactaatcattagttaataaggatacagatttattgactaatcatattaaataattaggtaagttaactattatttagttaagttagctaattgatttatatgattatcacaattgaatgtaatcataagcacaatcactgccatgtataatcacgggtatacgggtggttttcatatggggaacagggtgggcttagggccaggtcaccttaagtgacctttttttgtatatatggatgtagatttcaattgatcgaatactaatcctactgtcctcttttcttttcctttctccttctttactaacaacaacaaactacctcacaaccttctacctcaatatatactacctcattaagttgtttccttttgtctttttagggagcatactactacta

**SEOV-2010-China (GU592941)**

tagtagtaggctccctaaagagctactacactaacaagaaaaatggcaactatggaagaaatccagagagaaatcagtgctcacgaggggcagcttgtgatagcacgccagaaggtcaaggatgcagaaaagcagtatgagaaggatcctgatgacttaaacaagagggcactgcatgatcgggagagtgtcgcagcttcaatacaatcaaaaattgatgaattgaagcgccaacttgccgacaggattgcagcagggaagaacatcgggcaagaccgggatcctacaggggtagagccgggtgatcatctcaaggaaagatcagcactaagctatgggaatacactggacctgaatagtcttgacattgatgaacctacaggacagacagctgattggttgaccataattgtctatctgacatcattcgtggtcccaatcatcttgaaggcactgtacatgttaacaacaagaggtaggcagacttcaaaggacaacaaggggatgaggatcagattcaaggatgacagctcatatgaggatgtcaatggaatcagaaagcccaaacatctgtatgtgtcaatgccaaacgcccaatcaagcatgaaagctgaagagataacacctggaagattccgcacggcagtatgtgggttatatcctgcacagataaaggcaaggaacatggtaagccctgtcatgagtgtagttgggtttttggcactggcaaaagactggacatctagaattgaagaatggcttggcgcaccctgcaagttcatggcagagtctcccattgccgggagtttatctgggaatcctgtgaatcgtgactatatcagacagagacaaggtgcacttgcaggaatggagccaaaggaatttcaagccctcaggcaacatgcaaaggatgctggatgtacactggttgaacatattgagtcaccatcgtcaatatgggtgtttgctggggcccctgataggtgtccaccaacatgcttgtttgtcggagggatggctgagttaggtgccttcttttctatccttcaggacatgaggaacacaatcatggcttcaaaaactgtgggcacagctgatgaaaagcttcgaaagaaatcatcattctatcaatcatacctcagacgcacacaatcaatgggaatacaactggaccagaggataattgttatgtttatggtcgcctggggaaaggaagcagtggacaactttcatctcggtgatgacatggatccagagcttcgtagcctggctcagatcctgattgaccagaaagtgaaggaaatctctaaccaggaacctatgaaactataagtacataattatgtaatcaatactaactataggttaagaaatactaatcattagttaataaggatacagatttattgactaatcatattaaataattaggtaagttaactattatttagttaagttagctaattgatttatatgattatcacaattgaatgtaatcataagcacaatcactgccatgtataatcacgggtatacgggtggttttcatatggggaacagggtgggcttagggccaggtcaccttaagtgacctttttttgtatatatggatgtagatttcaattgatcgaatactaatcctactgtcctcttttcttttcctttctccttctttactaacaacaacaaactacctcacaaccttctacctcaatatatactacctcattaagttgtttccttttgtctctttagggagcatactactacta

**SEOV-2009-China (GQ279387)**

ccctaaagagctactacactaacaagaaaaatggcaactatggaagaaatccagagagaaatcagtgctcacgaggggcagcttgtgatagcacgccagaaggtcaaggatgcagaaaagcagtatgagaaggatcctgatgacttaaacaagagggcactgcatgatcgggagagtgttgcagcttcaatacaatcaaaaattgatgaattgaagcgccaacttgccgacaggattgcagcagggaagaacatcgggcaagaccgggatcctacaggggtagagccgggtgatcacctcaaggaaagatcagcactaagctatgggaatacactggacctgaatagtcttgacattgatgaacctacaggacagacagctgattggttgaccataattgtctatctgacatcattcgtggtcccaatcatcttgaaggcactgtacatgttaacaacaagaggtaggcagacttcaaaggacaacaaggggatgaggatcagattcaaggatgacagctcatatgaggatgtcaatggaatcagaaagcccaaacatctgtatgtgtcaatgccaaatgcccaatcaagcatgaaagctgaagagataacacctggaagattccgcacggcagtatgtgggttatatcctgcacagataaaggcaaggagcatggtaagccctgtcatgagtgtagttgggtttttggcactggcaaaagactggacatctagaattgaagaatggcttggcgcaccctgcaagttcatggcagagtctcccattgccgggagtttatctgggaatcctgtaaatcgtgactatatcagacagagacaaggtgcacttgcaggaatggagccaaaggaatttcaagccctcaggcaacatgcaaaggatgctggatgtacactggttgaacatattgagtcaccatcgtcaatatgggtgtttgctggggcccctgataggtgtccaccaacatgcttgtttgtcggagggatggctgaattaggtgccttcttttctatccttcaggacatgaggaacacaatcatggcttcaaaaactgtgggcacagctgatgaaaagcttcgaaagaaatcatcattctatcaatcatacctcagacgcacacaatcaatgggaatacaactggaccagaggataattgttatgtttatggttgcctggggaaaggaagcagtggacaactttcatctcggtgatgacatggatccagagcttcgtagcctggctcagatcctgattgaccagaaagtgaaggaaatctctaaccaggaacctatgaaactataagtacataattatgtaatcaatactaactataggttaagaaatactaatcattagttaataaggatacagatttattgactaatcatattaaataattaggtaagttaaccattatttagttaagttagctaattgatttatatgattatcacaattgaatgtaatcataagcacaatcactgccatgtataatcacgggtatacgggtggttttcatatggggaacagggtgggcttagggccaggtcaccttaagtgacctttttttgtatatatggatgtagatttcaattgatcgaatactaatcctactgtcctcttttcttttcctttctccttctttactaacaacaacaaactacctcacaaccttctacctcaatatatactacctcattaagttgtttccttttgtctttttaggga

**SEOV-2009-China (GQ279386)**

ccctaaagagctactacactaacaagaaaaatggcaactatggaagaaatccagagagaaatcagtgctcacgaggggcagcttgtgatagcacgccagaaggttaaggatgcagaaaagcagtatgagaaggatcctgatgacttaaacaagagggcactgcatgatcgggagagtgttgcagcttcaatacaatcaaaaattgatgaattgaagcgccaacttgccgacaggattgcagcagggaagaacatcgggcaagaccgggatcctacaggggtagagccgggtgatcacctcaaggaaagatcagcactaagctatgggaatacactggacctgaatagtcttgacattgatgaacctacaggacagacagctgattggttgaccataattgtctatctgacatcattcgtggtcccgattatcttgaaggcactgtacatgttaacagcaagaggtaggcagacttcaaaggacaacaaggggatgaggatcagattcaaggatgacagctcatatgaggatgtcaatggaatcagaaagcccaaacatctgtatgtgtcaatgccaaatgcccaatcaagcatgaaagctgaagagataacacctggaagattccgcacggcagtatgtgggttatatcctgcacagataaaggcaaggagcatggtaagccctgtcatgagtgtagttgggtttttggcactggcaaaagactggacatctagaattgaagaatggcttggcgcaccctgcaagttcatggcagagtctcccattgccgggagtttatctgggaatcctgtaaatcgtgactatatcagacagagacaaggtgcacttgcaggaatggagccaaaggaatttcaagccctcaggcaacatgcaaaggatgctggatgtacactggttgaacatattgagtcaccatcgtcaatatgggtgtttgctggggcccctgataggtgtccaccaacatgcttgtttgtcggagggatggctgaattaggtgccttcttttctatccttcaggacatgaggaacacaatcatggcttcaaaaactgtgggcacagctgatgaaaagcttcgaaagaaatcatcattctatcaatcatacctcagacgcacacaatcaatgggaatacaactggaccagaggataattgttatgtttatggttgcctggggaaaggaagcagtggacaactttcatctcggtgatgacatggatccagagcttcgtagcctggctcagatcctgattgaccagaaagtgaaggaaatctctaaccaggaacctatgaaactataagtacataattatgtaatcaatactaactataggttaagaaatactaatcattagttaataaggatacagatttattgactaatcatattaaataattaggtaagttaaccattatttagttaagttagctaattgatttatatgattatcacaattgaatgtaatcataagcacaatcactgccatgtataatcacgggtatacgggtggttttcatatggggaacagggtgggcttagggccaggtcaccttaagtgacctttttttgtatatatggatgtagatttcaattgatcgaatactaatcctactgtcctcttttcttttcctttctccttctttactaacaacaacaaactacctcacaaccttctacctcaatatatactacctcattaagttgtttccttttgtctttttagggag

**SEOV-2009-China (GQ279384)**

tagtagtaagctccctaaagagctactacactaacaagaaaaatggcaactatggaagaaatccagagagaaatcagtgctcacgaggggcagcttgtgatagcacgccagaaggtcaaggatgcagaaaaacagtatgagaaggatcctgatgacttaaacaagagggcactgcatgatcgggagagtgttgcagcttcaatacaatcaaaaattgatgaattgaagcgccaacttgccgacaggattgcagcagggaagaacatcgggcaagaccgggatcctacaggggtagagccgggtgatcatctcaaggaaagatcagcactaagctatgggaatacactggacctgaatagtcttgacattgatgaacctacaggacagacagctgattggttgaccataattgtctatctgacatcattcgtggtcccgatcatcttgaaggcactgtacatgttaacaacaagaggtaggcagacttcaaaggacaacaaggggatgaggatcagattcaaggatgacagctcatatgaggatgtcaatggaatcagaaagcccaaacatctgtatgtgtcaatgccaaatgcccaatcaagcatgaaagctgaagagataacacctggaagattccgcacggcagtatgtgggttatatcctgcacagataaaggcaaggaacatggtaagccctgtcatgagtgtagttgggtttttggcactggcaaaagactggacatctagaattgaagaatggcttggcgcaccctgcaagttcatggcagagtctcccattgccgggagtttatctgggaatcctgtaaatcgtgactatatcagacagagacaaggtgcacttgcaggaatggagccaaaggaatttcaagccctcaggcaacatgcaaaggatgctggatgtacactggttgaacatattgagtcaccatcgtcaatatgggtgtttgctggggcccctgataggtgtccaccaacatgcttgtttgtcggagggatggctgaattaggtgccttcttttctatccttcaggacatgaggaacacaatcatggcttcaaaaactgtgggcacagctgatgaaaagcttcgaaagaaatcatcattctatcaatcatacctcagacgcacacaatcaatgggaatacaactggaccagaggataattgttatgtttatggttgcctggggaaaggaagcagtggacaactttcatctcggtgatgacatggatccagagcttcgtagcctggctcagatcctgattgaccagaaagtgaaggaaatctctaaccaggaacctatgaaactataagtacataattatgtaatcaatactaactataggttaagaaatactaatcattagttaataaggatacagatttattgactaatcatattaaataattaggtaagttaaccattatttagttaagttagctaattgatttatatgattatcacaattgaatgtaatcataagcacaatcactgccatgtataatcacgggtatacgggtggttttcatatggggaacagggtgggcttagggccaggtcaccttaagtgacctttttttgtatatatggatgtagatttcaattgatcgaatactaatcctactgtcctcttttcttttcctttctccttctttactaacaacaacaaactacctcacaaccttctacctcaatatatactacctcattaagttgtttccttttgtctttttagg

**SEOV-2009-China (GQ279382)**

tagtagtagactccctaaagagctattacactaacaagaaaaatggcaactatggaagaaatccagagagaaatcagtgctcacgaggggcagcttgtgatagcacgccagaaggtcaaggatgcagaaaagcagtatgagaaggatcctgatgacttaaacaagagggcactgcatgatcgggagagtgttgcagcttcaatacaatcaaaaattgatgaattgaagcgccaacttgccgacaggattgcagcagggaagaacatcgggcaagaccgggatcctacaggggtagagccgggtgatcacctcaaggaaagatcagcactaagctatgggaatacactggacctgaatagtcttgacattgatgaacctacaggacagacagctgattggttgaccataattgtctatctgacatcattcgtggtcccaatcatcttgaaggcactgtacatgttaacaacaagaggtaggcagacttcaaaggacaacaaggggatgaggatcagattcaaggatgacagctcatatgaggatgtcaatggaatcagaaagcccaaacatctgtatgtgtcaatgccaaatgcccaatcaagcatgaaagctgaagagataacacctggaagattccgcacggcagtatgtgggttatatcctgcacagataaaggcaaggagcatggtaagccctgtcatgagtgtagttgggtttttggcactggcaaaagactggacatctagaattgaagaatggcttggcgcaccctgcaagttcatggcagagtctcccattgccgggagtttatctgggaatcctgtaaatcgtgactatatcagacagagacaaggtgcacttgcaggaatggagccaaaggaatttcaagccctcaggcaacatgcaaaggatgctggatgtacactggttgaacatattgagtcaccatcgtcaatatgggtgtttgctggggcccctgataggtgtccaccaacatgcttgtttgtcggagggatggctgaattaggtgccttcttttctatccttcaggacatgaggaacacaatcatggcttcaaaaactgtgggcacagctgatgaaaagcttcgaaagaaatcatcattctatcaatcatacctcagacgcacacaatcaatgggaatacaactggaccagaggataattgttatgtttatggttgcctggggaaaggaagcagtggacaactttcatctcggtgatgacatggatccagagcttcgtagcctggctcagatcctgattgaccagaaagtgaaggaaatctctaaccaggaacctatgaaactataagtacataattatgtaatcaatactaactataggttaagaaatactaatcattagttaataaggatacagatttattgactaatcatattaaataattaggtaagttaaccattatttagttaagttagctaattgatttatatgattatcacaattgaatgtaatcataagcacaatcactgccatgtataatcacgggtatacgggtggttttcatatggggaacagggtgggcttagggccaggtcaccttaagtgacctttttttgtatatatggatgtagatttcaattgatcgaatactaatcctactgtcctcttttcttttcctttctccttctttactaacaacaacaaactacctcacaaccttctacctcaatatatactacctcattaagttgtttccttttgtctttttagggagcatcttactac

**SEOV-2009-China (FJ803214)**

tagtagtaggctccctaaagagctactacactaacaagaaaaatggcaactatggaagaaatccagagagaaatcagtgctcacgaggggcagcttgtgatagcacgccagaaggtcaaggatgcagaaaagcagtatgagaaggatcctgatgacttaaacaagagggcactgcatgatcgggagagtgtcgcagcttcaatacaatcaaaaattgatgaattgaagcgccaacttgccgacaggattgcagcagggaagaacatcgggcaagaccgggatcctacaggggtagagccgggtgatcatctcaaggaaagatcagcactaagctatgggaatacactggacctgaatagtcttgacattgatgaacctacaggacagacagctgattggttgaccataattgtctatctgacatcattcgtggtcccaatcatcttgaaggcactgtacatgttaacaacaagaggtaggcagacttcaaaggacaacaaggggatgaggatcagattcaaggatgacagctcatatgaggatgtcaatggaatcagaaagcccaaacatctgtatgtgtcaatgccaaacgcccaatcaagcatgaaagctgaagagataacacctggaagattccgcacggcagtatgtgggttatatcctgcacagataaaggcaaggaacatggtaagccctgtcatgagtgtggttgggtttttggcactggcaaaagactggacatctagaattgaagaatggcttggcgcaccctgcaagttcatggcagagtctcccattgccgggagtttatctgggaatcctgtgaatcgtgactatatcagacagagacaaggtgcacttgcaggaatggagccaaaggaatttcaagccctcaggcaacatgcaaaggatgctggatgtacactggttgaacatattgagtcaccatcgtcaatatgggtgtttgctggggcccctgataggtgtccaccaacatgcttgtttgtcggagggatggctgagttaggtgccttcttttctatccttcaggacatgaggaacacaatcaaggcttcaaaaactgtgggcacagctgatgaaaagcttcgaaagaaatcatcattctatcaatcatacctcagacgcacacaatcaatgggaatacaactggaccagaggataattgttatgtttatggttgcctggggaaaggaagcagtggacaactttcatctcggtgatgacatggatccagagcttcgtagcctggctcagatcctgattgaccagaaagtgaaggaaatctctaaccaggaacctatgaaactataagtacataattatgtaatcaatactaactataggttaagaaatactaatcattagttaataaggatacagatttattgactaatcatattaaataattaggtaagttaactattatttagttaagttagctaattgatttatatgattatcacaattgaatgtaatcataagcacaatcactgccatgtataatcacgggtatacgggtggttttcatatggggaacagggtgggcttagggccaggtcaccttaagtgacctttttttgtatatatggatgtagatttcaattgatcgaatactaatcctactgtcctcttttcttttcctttctccttctttactaacaacaacaaactacctcacaaccttctacctcaatatatactacctcattaagttgtttccttttgtctctttagggagcatactactacta

**SEOV-2009-China (FJ803213)**

tagtagtaggctccctaaagagctactacactaacaagaaaaatggcaactatggaagaaatccagagagaaatcagtgctcacgaggggcagcttgtgatagcacgccagaaggtcaaggatgcagaaaagcagtatgagaaggatcctgatgacttaaacaagagggcactgcatgatcgggagagtgtcgcagcttcaatacaatcaaaaattgatgaattgaagcgccaacttgccgacaggattgcagcagggaagaacatcgggcaagaccgggatcctacaggggtagagccgggtgatcatctcaaggaaagatcagcactaagctatgggaatacactggacctgaatagtcttgacattgatgaacctacaggacagacagctgattggttgaccataattgtctatctgacatcattcgtggtcccaatcatcttgaaggcactgtacatgttaacaacaagaggtaggcagacttcaaaggacaacaaggggatgaggatcagattcaaggatgacagctcatatgaggatgtcaatggaatcagaaagcccaaacatctgtatgtgtcaatgccaaacgcccaatcaagcatgaaagctgaagagataacacctggaagattccgcatggcagtatgtgggttatatcctgcacagataaaggcaaggaacatggtaagccctgtcatgagtgtagttgggtttttggcactggcaaaagactggacatctagaattgaagaatggcttggcgcaccctgcaagttcatggcagagtctcccattgccgggagtttatctgggaatccggtgaatcgtgactatatcagacagagacaaggtgcacttgcaggaatggagccaaaggaatttcaagccctcaggcaacatgcaaaggatgctggatgtacactggttgaacatattgagtcaccatcgtcaatatgggtgtttgctggggcccctgataggtgtccaccaacatgcttgtttgtcggagggatggctgagttaggtgccttcttttctatccttcaggacatgaggaacacaatcatggcttcaaaaactgtgggcacagctgatgaaaagcttcgaaagaaatcatcattctatcaatcatacctcagacgcacacaatcaatgggaatacaactggaccagaggataattgttatgtttatggttgcctggggaaaggaagcagtggacaactttcatctcggtgatgacatagatccagagcttcgtagcctggctcagatcctgattgaccagaaagtgaaggaaatctctaaccaggaacctatgaaactataagtacataattatgtaatcaatactaactataggttaagaaatactaatcattagttaataaggatacagatttattgactaatcatattaaataattaggtaagttaactattatttagttaagttagctaattgatttatatgattatcacaattgaatgtaatcataagcacaatcactgccatgtataatcacgggtatacgggtggttttcatatggggaacagggtgggcttagggccaggtcaccttaagtgacctttttttgtatatatggatgtagatttcaattgatcgaatactaatcctactgtcctcttttcttttcctttctccttctttactaacaacaacaaactacctcacaaccttctacctcaatatatactacctcattaagttgtttccttttgtctttttagggagcatactactacta

**SEOV-2009-China (FJ803216)**

tagtagtaggctccctaaagagctactacactaacaagaaaaatggcaactatggaagaaatccagagagaaatcagtgctcacgaggggcagcttgtgatagcacgccagaaggtcaaggatgcagaaaagcagtatgagaaggatcctgatgacttaaacaagagggcactgcatgatcgggagagtgtcgcagcttcaatacaatcaaaaattgatgaattgaagcgccaacttgccgacaggattgcagcagggaagaacatcgggcaagaccgggatcctacaggggtagagccgggtgatcatctcaaggaaagatcagcactaagctatgggaatacactggacctgaatagtcttgacattgatgaacctacaggacagacagctgattggttgaccataattgtctatctgacatcattcgtggtcccaatcatcttgaaggcactgtacatgttaacaacaagaggtaggcagacttcaaaggacaacaaggggatgaggatcagattcaaggatgacagctcatatgaggatgtcaatggaatcagaaagcccaaacatctgtatgtgtcaatgccaaacgcccaatcaagcatgaaagctgaagagataacacctggaagattccgcacggcagtatgtgggttatatcctgcacagataaaggcaaggaacatggtaagccctgtcatgagtgtggttgggtttttggcactggcaaaagactggacatctagaattgaagaatggcttggcgcaccctgcaagttcatggcagagtctcccattgccgggagtttatctgggaatcctgtgaatcgtgactatatcagacagagacaaggtgcacttgcaggaatggagccaaaggaatttcaagccctcaggcaacatgcaaaggatgctggatgtacactggttgaacatattgagtcaccatcgtcaatatgggtgtttgctggggcccctgataggtgtccaccaacatgcttgtttgtcggagggatggctgagttaggtgccttcttttctatccttcaggacatgaggaacacaatcatggcttcaaaaactgtgggcacagctgatgaaaagcttcgaaagaaatcatcattctatcaatcatacctcagacgcacacaatcaatgggaatacaactggaccagaggataattgttatgtttatggtcgcctggggaaaggaagcagtggacaactttcatctcggtgatgacatggatccagagcttcgtagcctggctcagatcctgattgaccagaaagtgaaggaaatctctaaccaggaacctatgaaactataagtacataattatgtaatcaatactaactataggttaagaaatactaatcattagttaataaggatacagatttattgactaatcatattaaataattaggtaagttaactattatttagttaagttagctaattgatttatatgattatcacaattgaatgtaatcataagcacaatcactgccatgtataatcacgggtatacgggtggttttcatatggggaacagggtgggcttagggccaggtcaccttaagtgacctttttttgtatatatggatgtagatttcaattgatcgaatactaatcctactgtcctcttttcttttcctttctccttctttactaacaacaacaaactacctcacaaccttctacctcaatatatactacctcattaagttgtttccttttgtctctttagggagcatactactacta

**HTNV-2017-China (KY639701)**

tagtagtaggctccctaaagagctactatactaacaagaaaaatggcaactatggaagaaatccagagagaaatcagtgctcacgaggggcagcttgtgatagcacgccagaaggttaaggatgcagaaaagcagtatgagaaggatcctgatgacttaaacaagagggcactgcatgatcgggagagtgtcgcagcttcaatacaatcaaaaattgatgaattgaagcgccaacttgccgacaggattgcagcaggaaaaaacatcgggcaagaccgggatcctacaggggtagagccgggtgatcatctcaaggaaagatcagcactaagctatgggaatacactggacctgaatagtcttgacattgatgaacctacaggacagacagctgattggttgaccataattgtctatctgacatcattcgtggtcccgatcattttgaaggcactgtatatgttaacaacaagaggtaggcagacttcaaaggacaacaaggggatgaggatcagattcaaggatgacagctcatatgaggatgtcaatgggatcagaaagcccaaacatctgtatgtgtcaatgccaaacgcccaatccagcatgaaagctgaagagataacacctggaagattccgtacggcagtatgtgggctatatcctgcacagataaaggcaagaaacatggtaagccctgtcatgagtgttgttgggtttttggcactggcaaaagactggacatctagaattgaagaatggcttggcgcaccttgcaagttcatggcggagtctcccattgccgggagtttatctgggactcctgtgaatcgtgactatatcagacagagacaaggtgcacttgcaggaatggagccaaaggaatttcaagccctcaggcaacatgcaaaggatgctggatgtacactggttgaacatattgagtcaccatcgtcaatatgggtgtttgctggggcccctgataggtgtccaccaacatgcttgtttgtcggaggtatggctgagttaggtgccttcttttctatccttcaggatatgaggaacacaatcatggcttcaaaaactgtgggcacagctgatgaaaagcttcgaaagaaatcatcattctatcaatcatacctcagacgcacacaatcaatgggaatacaactggaccagaggataattgttatgtttatggttgcctggggaaaggaggcagtggacaactttcatctcggtgatgacatggatccagagcttcgtagcctggctcagatcctgattgaccagaaagtgaaggaaatctcaaaccaggaacctatgaaattataagtacataattatgtaatcaatactaactataggttaagaaatactaatcattagttaataagaatacagatttattgaataatcatattaaataattaggtaagttaactattatttagttaagttagctaattgatttatatgattatcacaattgaatgtaatcataagcacaatcactgccatgtataatcacgggtatacgggtggttttcatatggggaacagggtgggcttagagccaggtcaccttaagtgacctttttttgtatatatggatgtagatttcaattgatcgaatactaattctactgtcctcttttcttttcctttctccttctttactaacaacaacaaactacctcacaacattctacctcaatatatactacctcattaagttgtttccttttgtctttttagggagcatactactacta

**HTNV-2017-China (KY639689)**

ggcccctaaagagctactacactaacaagaaaaatggcaactatggaagaaatccagagagaaatcagtgctcacgaggggcagcttgtgatagcacgccagaaggttaaggatgcagaaaagcagtatgagaaggatcctgatgacttaaacaagagggcactgcatgatcgggagagtgtcgcagcttcaatacaatcaaaaattgatgaattgaagcgccaacttgccgacaggattgcagcaggaaaaaacatcgggcaagaccgggatcctacaggggtagagccgggtgatcatctcaaggaaagatcagcactaagctatgggaatacactggacctgaatagtcttgacattgatgaacctacaggacagacagctgattggttgaccataattgtctatctgacatcattcgtggtcccgatcattttgaaggcactgtacatgttaacaacaagaggtaggcagacttcaaaggacaacaaggggatgaggatcagattcaaggatgacagctcatatgaggatgtcaatggaatcagaaagcccaaacatctgtatgtgtcaatgccaaacgcccaatccagcatgaaagctgaagagataacacctggaagattccgtacggcagtatgtgggctatatcctgcacagataaaggcaagaaacatggtaagccctgtcatgagtgtagttgggtttttggcactggcaaaagactggacatctagaattgaagaatggcttggcgcaccctgcaagtttatggcagagtctcccattgccgggagtttatctgggaatcctgtgaatcgtgactatatcagacagagacaaggtgcacttgcaggaatggagccaaaggaatttcaagcccttaggcaacatgcaaaggatgctggatgtacactggttgaacatattgagtcaccatcgtcaatatgggtgtttgctggggcccctgataggtgtccaccaacatgcttgtttgtcggaggtatggctgagttaggtgccttcttttctatccttcaggatatgaggaacacaatcatggcttcgaaaactgtgggcacagctgatgaaaagcttcgaaagaaatcatcattctatcaatcatacctcagacgcacacaatcaatgggaatacaactggaccagaggataattgttatgtttatggttgcctggggaaaggaggcagtggacaactttcatctcggtgatgacatggatccagagcttcgtagcctggctcagatcctgattgaccagaaagtgaaggaaatctcaaaccaggaacctatgaaattataagtacataattatgtaatcaatactaactataggttaagagatactaatcattagtcaataagaatacagatttattgaataatcatattaaatgattaggtaagttaactattatttagttaagttagctaattgatttatatgattatcacaattaaatgtaatcataagcacaatcactgccatgtataatcacgggtatacgggtggttttcatatggggaacagggtgggcttagggccaggtcaccttaagtgacctttttttgtatatatggatgtagatttcaattgatcgaatactaatcctactgtcctcttttcttttcctttctccttctttactaacaacaacaaactacctcacaaccttctacctcaatatatactacctcattaagttgtttccttttgtctctttagggagcatactactacta

**HTNV-2017-China (KY639683)**

tagtagtaggctccgtaaagagctagtacactaacaagaaaaatggcaactatggaagaaatccagagagaaatcagtgctcacgaggggcagcttgtgatagcacgccagaaggttaaggatgcagaaaagcagtatgagaaggatcctgatgacttaaacaagagggcactgcatgatcgggagagtgtcgcagcttcaatacaatcaaaaattgatgaattgaagcgccaacttgccgacaggattgcagcaggaaaaaacatcgggcaagaccgggatcctacaggggtagagccgggtgatcatctcaaagaaagatcagcactaagctatgggaatacactggacctgaatagtcttgacattgatgaacctacaggacagacagctgattggttgaccataattgtctatctgacatcattcgtggtcccgatcattttgaaggcattgtacatgttaacaacaagaggtaggcagacttcaaaggacaacaaggggatgaggatcagattcaaggatgacagctcatatgaggatgtcaatgggatcagaaagcccaaacatctgtatgtgtcaatgccaaacgcccaatccagcatgaaagctgaagagataacacctggaagattccgtacggcagtatgtgggctatatcctgcacagataaaggcaagaaacatggtaagccctgtcatgagtgttgttgggtttttggcactggcaaaagactggacatctagaattgaagaatggcttggcgcaccttgcaagttcatggcggagtctcccattgccgggagtttatctgggactcctgtgaatcgtgactatatcagacagagacaaggtgcacttgcaggaatggagccaaaggaatttcaagccctcaggcaacatgcaaaggatgctggatgtacactggttgaacatattgagtcaccatcgtcaatatgggtgtttgctggggcccctgataggtgtccaccaacatgcttgtttgttggaggtatggctgagttaggtgccttcttttctatccttcaggatatgaggaacacaatcatggcttcaaaaactgtgggcacagctgatgaaaagcttcgaaagaaatcatcattctatcaatcatacctcagacgcacacaatcaatgggaatacaactggaccagaggataattgttatgtttatggttgcctggggaaaggaggcagtggacaactttcatctcggtgatgacatggatccagagcttcgtagcctggctcagatcctgattgaccagaaagtgaaggaaatctcaaaccaggaacctatgaaattataagtacataattatgtaatcaatactaactataggttaagaaatactaatcattagttaataagaatacagatttattgaataatcatattaaataattaggtaagttaactattatttagttaagttagctaattgatttatatgattatcacaattgaatgtaatcataagcacaatcactgccatgtataatcacgggtatacgggtggttttcatatggggaacagggtgggcttagggccaggtcaccttaagtgacctttttttgtatatatggatgtagatttcaattgatcgaatactaatcctactgtcctcttttcttttcctttctccttctttactaacaacaacaaactacctcacaaccttctacctcaatatatactacctcattaagttgtttccttttgtctttttagggagcatactactactacta

**HTNV-2017-China (KY639679)**

tagtagtaggctccctaaagagctactacactaacaagaaaaatggcaactatggaagaaatccagagagaaatcagtgctcacgaggggcagcttgtgatagcacgccagaaggttaaggatgcagaaaagcagtatgagaaggatcctgatgacttaaacaagagggcactgcatgatcgggagagtgttgcagcttcaatacaatcaaaaattgatgaattgaagcgccaacttgccgacaggattgcagcaggaaaaaacatcgggcaagaccgggatcctacaggggtagagccgggtgatcatctcaaggaaagatcagcactaagctatgggaatacactggacctgaatagtcttgacattgatgaacctacaggacagacagctgattggttgaccataattgtctatctgacatcattcgtggtcccgatcattttgaaggcactgtacatgttaacaacaagaggcaggcagacttcaaaggacaacaaggggatgaggatcagattcaaggatgacagctcatatgaggatgtcaatgggatcagaaagcccaaacatctgtatgtgtcaatgccaaatgcccaatccagcatgaaagctgaagagataacacctggaagattccgtacggcagtatgtgggctatatcctgcacagataaaggcaagaaacatggtaagccctgtcatgagtgttgttgggtttttggcactggcaaaagactggacatctagaattgaagaatggcttggcgcaccttgcaagttcatggcggagtctcccattgccgggagtttatctgggactcctgtgaatcgtgactatatcagacagagacaaggtgcacttgcaggaatggagccaaaggaatttcaagccctcaggcaacatgcaaaggatgctggatgtacactggttgaacatattgagtcaccatcgtcaatatgggtgtttgctggggcccctgataggtgtccaccaacatgtctgtttgtcggaggtatggctgagttaggtgccttcttttctatccttcaggatatgaggaacacaatcatggcttcaaaaactgtgggcacagctgatgaaaagcttcgaaagaaatcatcattctatcaatcatacctcagacgcacacaatcaatgggaatacaactggaccagaggataattgttatgtttatggttgcctggggaaaggaggcagtggacaactttcatctcggtgatgacatggatccagagcttcgtatcctggctcagatcctgattgaccagaaagtgaaggaaatctcaaaccaggaacctatgaaattataagtacataattaggtaatcaatactaactataggttaagaaatactaatcattagttaataagaatacagatttattgaataatcatattaaataattaggtaagttaactattatttagttaagttagctaattgatttatatgattatcacaattgaatgtaatcataagcacaatcactgccatgtataatcacgggtatacgggtggttttcatatggggaacagggtgggcttagggccaggtcaccttaagtgacctttttttgtatatatggatgtagatttcaattgatcgaatactaatcctactgtcctcttttcttttcctttctccttctttactaacaacaacaaactacctcacaaccttctacctcaatatatactacctcattaagttgtttccttttgtctctttagggagcatactactacta

**HTNV-2017-China (KY639676)**

tagtagtagactccctaaagagctactacactaacaagaaaaatggcaactatggaagaaatccagagagaaatcagtgctcacgaggggcagcttgtgatagcacgccagaaggttaaggatgcagaaaaacagtatgagaaggatcctgatgacttaaacaagagggcactgcatgatcgggagagtgtcgcagcttcaatacaatcaaaaattgatgaattgaagcgccaacttgccgacaggattgcagcaggaaaaaacattgggcaagaccgggatcctacaggggtagagccgggtgatcatctcaaagaaagatcagcactaagctatgggaatacactggacctgaatagtcttgacattgatgaacctacaggacagacagctgattggttgaccataattgtctatctgacatcattcgtggtcccgatcattttgaaggcattgtacatgttaacaacaagaggcaggcagacttcaaaggacaacaaggggatgaggatcagattcaaggatgacagctcatatgaggatgtcaatgggatcagaaagcccaaacatctgtatgtgtcaatgccaaatgcccaatccagcatgaaagctgaagagataacacctggaagattccgtacggcagtatgtgggctatatcctgcacagataaaggcaagaaacatggtaagccctgtcatgagtgttgttgggtttttggcactggcaaaagactggacatctagaattgaagaatggcttggcgcaccttgcaagttcatggcggagtctcccattgccgggagtttatctgggactcctgtgaatcgtgactatatcagacagagacaaggtgcacttgcaggaatggagccaaaggaatttcaagccctcaggcaacatgcaaaggatgctggatgtacactggttgaacatattgagtcaccatcgtcaatatgggtgtttgctggggcccctgataggtgtccaccaacatgtttgtttgtcggaggtatggctgagttaggtgccttcttttctatccttcaggatatgaggaacacaatcatggcttcaaaaactgtgggcacagctgatgaaaagcttcgaaagaaatcatcattctatcaatcatacctcagacgcacacaatcaatgggaatacaactggaccagaggataattgttatgtttatggttgcctggggaaaggaggcagtggacaactttcatctcggtgatgacatggatccagagctccgtagcctggctcagatcctgattgaccagaaagtgaaggaaatctcaaaccaggaacctatgaaattataagtacataattatgtaatcaatactaactataggttaagaaatactaatcattagttaataagaatacagatttattgaataatcatattaaataattaggtaagttaaccattatttagttaagttagctaattgatttatatgattatcacaattgaatgtaatcataagcacaatcactgccatgtataatcacgggtatacgggtggttttcatatggggaacagggtgggcttagggccaggtcaccttaagtgacctttttttgtatatatggatgtagatttcaattgatcgaatactaatcctactgtcctcttttcttttcctttctccttctttactaacaacaacaaactacctcacaaccttctacctcaatatatactacctcattaagttgtttccttttgtctttttagggagcatactactacta

**HTNV-2017-China (KY639673)**

tagtagttaggctccctaaagagctactacactaacaagaaaaatggcaactatggaagaaatccagagagaaatcagtgctcacgaggggcagcttgtgatagcacgccagaaggtcaaggatgcagaaaagcagtatgagaaggatcctgatgacttaaacaagagggcactgcatgatcgagagagtgtcgcagcttcaatacaatcaaaaattgatgaattgaagcgccaacttgccgacaggattgcagcaggaaagaacatcgggcaagaccgggatcctacaggggtagagccaggtgatcatctcaaggaaagatcagcactaagctatgggaatacactggacctgaatagtcttgacattgatgaacctacaggacagacagctgattggttgaccataattgtctatctgacatcatttgtggtcccgatcatcttgaaggcactgtacatgttaacaacaagaggcaggcagacttcaaaagacaacaaggggatgaggatcagattcaaggatgacagctcatatgaggatgtcaatggaatcaggaagcccaaacatctgtatgtgtcaatgccaaacgcccaatccagcatgaaagctgaagagataacacctggaagattccgcacggcagtatgtgggctatatcctgcacagataaaggcaaggaacatggtaagccctgtcatgagtgtagttgggtttttggcactggcaaaagactggacatctagaattgaagaatggcttggcgcaccctgcaagttcatggcggagtctcccattgccgggagtttatctgggaatcctgtgaatcgtgactatatcagacagagacaaggtgcacttgcaggaatggagccaaaggaatttcaagccctcaggcaacatgcaaaggatgctggatgtacactggttgaacatattgagtcaccatcatcaatatgggtgtttgctggggcccctgataggtgtccaccaacatgcttgtttgttggagggatggctgagttaggtgccttcttttctatccttcaggatatgaggaacacaatcatggcttcaaaaactgtgggcacagctgatgaaaagcttcgaaagaaatcatcattctatcaatcatacctcagacgcacacaatcaatgggaatacaactggaccagaggataattgttatgtttatggttgcctggggaaaggaggcagtggacaactttcatctcggtgatgacatggatccagagcttcgtagcctggctcagatcctgattgaccagaaagtgaaggagatctcaaaccaggaacctatgaaattataagtacatgactatgtaatcaatactaactataggttaagaaatactaatcattagttaataagaatacagatttattgaataatcatattaaataattaggtaagttaactattatttagttaagttagctaattgatttatattattatcacaattgaatgtaatcataagcacaatcactgccatgtataatcacgggtatacgggtggttttcatatggggaacagggtgggcttagggccaggtcaccttaagtgacctttttttgtatatatggatgtagatttcaattgatcgaatactaatcctactgtcctcttttcttttcctttctccttctttactaacaacaacaaactacctcacaaccttctacctcaatatatactacctcattaagttgtttccttttgtctttttagggagcatactactacta

**HTNV-2017-China (KY639672)**

tagtaggtaggtccctaaagagctactacactaacaagaaaaatggcaactatggaagaaatccagagagaaatcagtgctcacgaggggcagcttgtgatagcacgccagaaggttaaggatgccgaaaagcagtatgagaaggatcctgatgacttaaacaagagggcactgcatgatcgagagagtgtcgcagcttcaatacaatcaaaaattgatgaattgaagcgccaacttgctgacaggattgcagcagggaaaaacatcgggcaagaccgggatcctacaggggtagagccgggtgatcatctcaaggaaagatcagcactaagctatgggaatacactggacctgaatagtcttgacattgatgaacctacaggacagacagctgattggttgaccataattgtctatctgacatcattcgtggtcccgatcattttgaaggcactgtacatgttaacaacaagaggtaggcagacttcaaaggacaacaaggggatgaggatcagattcaaggatgacagctcatatgaggatgtcaatggaatcagaaagcccaaacatctgtatgtgtcaatgccaaacgcccaatccagcatgaaagctgaagagataacacctggaagattccgtacggcagtatgtgggctatatcctgcacagataaaggcaagaaacatggtaagccctgtcatgagtgtagttgggtttttggcactggcaaaagactggacatctagaattgaagaatggctcggcgcaccctgcaagttcatggcggagtctcccattgccgggagtttatctgggaatcctgtgaatcgtgactatatcagacagagacaaggtgcacttgcaggaatggagccaaaggaatttcaagccctcaggcaacatgcaaaggatgctggatgtacactggttgaacatattgagtcaccatcgtcaatatgggtgtttgctggggcccctgataggtgtccaccaacatgcttgtttgtcggaggtatggctgagttaggtgccttcttttctatccttcaggatatgaggaacacaatcatggcttcaaaaactgtgggcacagctgatgaaaagcttcgaaagaaatcatcattctatcaatcatacctcagacgcacacaatcaatgggaatacaactggaccagaggataattgttatgtttatggttgcctggggaaaggaggcagtggacaactttcatctcggtgatgacatggatccagagcttcgtagcctggctcagatcctgattgaccagaaagtgaaggaaatctcaaaccaggaacctatgaaattataagtacataattatgtaatcaatactaactataggttaagaaatactaatcattagttaataagaatacagatttattgaataatcatattaaataattaagtaagttaactattatttagttaagttagctaattgatttatatgattatcacaattgaatgtaatcataagcacaatcactgccatgtataatcacgggtatacgggtggttttcatatggggaacagggtgggcttagggccaggtcaccttaagtgacctttttttgtatatatggatgtagatttcaattgatcgaatactaatcctactgtcctcttttcttttcctttctccttctttactaacaacaacaaactacctcacaaccttctacctcaatatatactacctcattaagttgtttccttttgtctttttagggagcatactactacta

**HTNV-2017-China (KY639670)**

tagtagttaggtccctaaagagctactacactaacaagaaaaatggcaactatggaagaaatccagagagaaatcagtgctcacgaggggcagcttgtgatagcacgccagaaggttaaggatgcagaaaagcagtatgagaaggatcctgatgacttaaacaagagggcactgcatgatcgggagagtgtcgcagcttcaatacaatcaaaaattgatgaattgaagcgccaacttgccgacaggattgcagcaggaaaaaacatcgggcaagaccgggatcctacaggggtagagccgggtgatcatctcaaggaaagatcagcactaagctatggaaatacactggacctgaatagtcttgacattgatgaacctacaggacagacagctgattggttgaccataattgtctatctgacatcattcgtggtcccaatcattttgaaggcactgtacatgttaacaacaagaggtaggcagacttcaaaggacaacaaggggatgaggatcagattcaaggatgacagctcatatgaggatgtcaatgggatcagaaagcccaaacatctgtatgtgtcaatgccaaacgcccaatccagcatgaaagctgaagagataacacctggaagattccgtacggcagtatgtgggctatatcctgcacagataaaggcaagaaacatggtaagccctgtcatgagtgttgttgggtttttggcactggcaaaagactggacatctagaattgaagaatggcttggcgcaccttgcaagttcatggcggagtctcccattgccgggagtttatctgggactcctgtgaatcgtgactatatcagacagagacaaggtgcacttgcaggaatggagccaaagaatttcaagccctcaggcaacatgcaaaggatgctggatgtacactggttgaacatattgagtcaccatcgtcaatatgggtgtttgctggggcccctgataggtgtccaccaacatgcttgtttgttggaggtatggctgagttaggtgccttcttttctatccttcaggatatgaggaacacaatcatggcttcaaaaactgtgggcacagctgatgaaaagcttcgaaagaaatcatcattctatcaatcatacctcagacgcacacaatcaatgggaatacaactggaccagaggataattgttatgtttatggttgcctggggaaaggaggcagtggacaactttcatctcggtgatgacatggatccagagcttcgtagcctggctcagatcctgattgaccagaaagtgaaggaaatctcaaaccaggaacctatgaaattataagtacataattatgtaatcaatactaactataggttaagaaatactaatcattagttaataagaatacagatttattgaataatcatattaaataattaggtaagttaactattatttagttaagttagctaattgatttatatgattatcacaattgaatgtaatcataagcacaatcactgccatgtataatcacgggtatacgggtggttttcatatggggaacagggtgggcttagggccaggtcaccttaagtgaccttttttttgtatatatggatgtagatttcaattgatcgaatactaatcctactgtcctcttttcttttcctttttccttctttactaacaacaacaaactacctcacaaccttctacctcaatatatactacctcattaagttgtttccttttgtctttttagggagcatactactacta

**HTNV-2017-China (KY639639)**

tagtagttaggtccctaaagagctactacactaacaagaaaaatggcaactatggaagaaatccagagagaaatcagtgctcacgaggggcagcttgtgatagcacgccagaaggttaaggatgcagaaaagcagtatgagaaggatcctgatgacttaaacaagagggcactgcatgatcgggagagtgtcgcagcttcaatacaatcaaaaattgatgaattgaagcgccaacttgccgacaggattgcagcaggaaaaaacatcgggcaagaccgggatcctacaggggtagagccgggtgatcatctcaaggaaagatcagcactaagctatggaaatacactggacctgaatagtcttgacattgatgaacctacaggacagacagctgattggttgaccataattgtctatctgacatcattcgtggtcccaatcattttgaaggcactgtacatgttaacaacaagaggtaggcagacttcaaaggacaacaaggggatgaggatcagattcaaggatgacagctcatatgaggatgtcaatgggatcagaaagcccaaacatctgtatgtgtcaatgccaaacgcccaatccagcatgaaagctgaagagataacacctggaagattccgtacggcagtatgtgggctatatcctgcacagataaaggcaagaaacatggtaagccctgtcatgagtgttgttgggtttttggcactggcaaaagactggacatctagaattgaagaatggcttggcgcaccttgcaagttcatggcggagtctcccattgccgggagtttatctgggactcctgtgaatcgtgactatatcagacagagacaaggtgcacttgcaggaatggagccaaaggaatttcaagccctcaggcaacatgcaaaggatgctggatgtacactggttgaacatattgagtcaccatcgtcaatatgggtgtttgctggggcccctgataggtgtccaccaacatgcttgtttgttggaggtatggctgagttaggtgccttcttttctatccttcaggatatgaggaacacaatcatggcttcaaaaactgtgggcacagctgatgaaaagcttcgaaagaaatcatcattctatcaatcatacctcagacgcacacaatcaatgggaatacaactggaccagaggataattgttatgtttatggttgcctggggaaaggaggcagtggacaactttcatctcggtgatgacatggatccagagcttcgtagcctggctcagatcctgattgaccagaaagtgaaggaaatctcaaaccaggaacctatgaaattataagtacataattatgtaatcaatactaactataggttaagaaatactaatcattagttaataagaatacagatttattgaataatcatattaaataattaggtaagttaactattatttagttaagttagctaattgatttatatgattatcacaattgaatgtaatcataagcacaatcactgccatgtataatcacgggtatacgggtggttttcatatggggaacagggtgggcttagggccaggtcaccttaagtgaccttttttttgtatatatggatgtagatttcaattgatcgaatactaatcctactgtcctcttttcttttcctttttccttctttactaacaacaacaaactacctcacaaccttctacctcaatatatactacctcattaagttgtttccttttgtctttttagggagcatactactacta

**HTNV-2004-China (AY627049)**

tagtagtagactccctaaagagctactacactaacaagaaaaatggcaactatggaagaaatccagagagaaatcagtgctcacgaggggcagcttgtgatagcacgccagaaggtcaaggatgcagaaaagcagtatgagaaggatcctgatgacttaaacaagagggcactgcatgatcgggagagtgttgcagcttcaatacaatcaaaaattgatgaattgaagcgccaacttgccgacaggattgcagcagggaagaacatcgggcaagaccgggatcctacaggggtagagccgggtgatcatctcaaggaaagatcagcactaagctatgggaatacactggacctgaatagtcttgacattgatgaacctacaggacagacagctgattggttgaccataattgtctatctgacatcattcgtggtcccaatcatcttgaaggcactgtacatgttaacaacaagaggtaggcagacttcaaaggataacaaggggatgaggatcagattcaaggatgacagctcatatgaggatgtcaatggaatcagaaagcccaaacatctgtatgtgtcaatgccaaatgcccaatcaagcatgaaagctgaagagataacacctggaagattccgcacggcagtatgtgggttatatcctgcacagataaaggcaaggaacatggtaagccctgtcatgagtgtagttgggtttttggcactggcaaaagactggacatctagaattgaagaatggcttggcgcaccctgcaagttcatggcagagtctcccattgccgggagtttatctgggaatcctgtaaatcgtgactatatcagacagagacaaggtgcacttgcaggaatggagccaaaggaatttcaagccctcaggcaacatgcaaaggatgctggatgtacactggttgaacatattgagtcaccatcgtcaatatgggtgtttgctggggcccctgataggtgtccaccaacatgcttgtttgtcggagggatggctgaattaggtgccttcttttctatccttcaggacatgaggaacacaatcatggcttcaaaaactgtgggcacagctgatgaaaagcttcgaaagaaatcatcattctatcaatcatacctcagacgcacacaatcaatgggaatacaactggaccagaggataattgttatgtttatggttgcctggggaaaggaagcagtggacaactttcatctcggtgatgacatggatccagagcttcgtagcctggctcagatcctgattgaccagaaagtgaaggaaatctctaaccaggaacctatgaaactataagtacatagttatgtaatcaatactaactataggttaagaaatactaatcattagttaagaaggatacagatttattgactaatcatattaaataattaggtaagttaaccattatttagttaagttagctaattgatttatatgattatcacaattgaatgtaatcataagcacaatcactgccatgtataatcacgggtacacgggtggttttcatatggggaacagggtgggcttagggccaggtcaccttaagtgacctttttttgtatatatggatgtagatttcaattgatcgaatactaatcctactgtcctcttttcttttcctttctccttctttactaacaacaacaaactacctcacaaccttctacctcaatatatactacctcattaagttgtttccttttgtctttttagggagcatactactacta
